# Internal and External Resources as Determinants of Health and Quality of Life

**DOI:** 10.1371/journal.pone.0153232

**Published:** 2016-05-02

**Authors:** Elfriede Greimel, Yoshiko Kato, Maria Müller-Gartner, Beate Salchinger, Roswith Roth, Wolfgang Freidl

**Affiliations:** 1 Medical University Graz, Graz, Austria; 2 Kobe University, Kobe, Japan; 3 FH JOANNEUM University of Applied Sciences, Graz, Austria; 4 Karl Franzens University Graz, Graz, Austria; National Cancer Center, JAPAN

## Abstract

**Background:**

The salutogenic model has been established as a health promoting resource that is related to a strong sense of coherence (SOC), positive subjective health and quality of life (QoL). The aim of the study was to compare internal and external resources, life style factors, perceived health and QoL in Japan and Austria and to determine associations among these factors.

**Methodology and Principal Findings:**

A survey was conducted in a Japanese (N = 460) and an Austrian (N = 421) student sample using the following self-report health questionnaires: Sense of Coherence Scale (SOC-13), Social and Gender Role Scale, Multidimensional Scale of Perceived Social Support (MSPSS), Dutch Eating Behaviour Questionnaire (DEBQ), SF-12 Health Survey, and the Cross-cultural Health Survey. Analyses of data showed that age (ß -0.12), and stress (ß -0.21) were negatively related and SOC (ß 0.47), family support are (ß -0.35) positively related to mental QoL. Significant predictors for emotional strain, were female gender (ß -0.24), older age (ß-0.14), lower SOC (ß 0.28), less traditional gender and social role patterns (ß 0.10), more restrained eating (ß -0.20), more alcohol intake (ß -0.16), and more stress (ß -0.25) explaining 42% of the variance in Austrian students. In Japan stress (ß -0.38) was negatively related and SOC (ß 0.37) positively related to mental QoL. Older age (ß -0.20), lower SOC (ß 0.29) and more stress (ß -0.33) were identified as significant predictors explaining 35% of the variance in Japanese students.

**Conclusions and Significance:**

SOC and stress are strongly associated with QoL and perceived health in Austria as well as in Japan. SOC seems to be a crucial predictor for stress, and emotional health independent of the cultural context. A major challenge of cross-cultural research is to understand perceived health and QoL and the extent in which it is individually, socially, or culturally determined.

## Introduction

About 30 years ago Antonovsky [1] introduced the salutogenic concept of health, indicating a change from a pathogenic to a salutogenic perspective. The pathological orientation limits health to medical characteristics and normative functioning of biological systems [2]. The salutogenic approach considers different dimensions of well-being as determinants of health within individuals and societies. This perspective is related to the WHO definition of health [3] and quality of life (QoL). This is defined as an “individuals’ perception of their position in life in the context of the culture and value systems in which they live and in relation to their goals, expectations, standards and concerns” [4]. Health and QoL is shaped by factors such as education, social networks, employment and working conditions, or health behaviour. The cultural background determines an individuals’ perception of health, meaning of QoL and well-being, experience of symptoms and distress, health behaviour patterns, emotional experiences, and cognitive appraisal. Culture is ‘a dynamic system of explicit and implicit rules established by groups in order to ensure their survival, involving attitudes, values, beliefs, norms and behaviours, shared by a group’ [5].

A cross-cultural comparison of health indicators across Europe showed that better health is related to higher education, employment, use of preventive medical services, better mental health, regular exercise, lower alcohol consumption and higher QoL. Poorer health is associated with greater age, and presence of chronic conditions [6]. In Eastern European countries the level of physical activity, overall QoL and the amount of social support were significantly lower than in Western European countries as well as lifetime prevalence of chronic conditions is significantly higher. Yu et al. [7] found in a sample of Chinese elderly that cultural factors specified by family relations, along with demographic factors, number of diseases, economic well-being, and living conditions have a significant impact on their subjective health status.

The salutogenic model stresses the strengths of individuals and their capacity for adjustment and explains why certain people seem to preserve health and successfully cope with the exposures to life stressors from their environment. Sense of coherence (SOC) is a central concept within the salutogenic model and a necessary condition for health and QoL. It is a global orientation to view the world and interact with the environment in a comprehensive, manageable, and meaningful way [8]. Sense of coherence is developed during childhood, adolescence, and through early adulthood. It is a stable disposition of personality and a health promoting resource that improves resilience and develops a positive state of both, physical and emotional well-being. The extent to which these resources are available is a major determinant in the development of a strong or weak SOC. A systematic review showed that SOC seems to have an impact on QoL [9]. A relationship between SOC, health and QoL was found in various clinical samples [10–14], general populations [15,16], and children [17]. The salutogenic model has also been applied within University settings including different student samples [18,19]. University attendance is regarded as a positive event that provides opportunities for individual development. It represents a critical development, in which students enter a new social environment where they have to adjust to new social norms and establish new relationships [20]. This experience is also accompanied by significant changes, stress, and challenges in academic, social, and emotional areas. University students may perceive academic life as stressful and demanding [21]. They report experiencing emotional reactions to this stress, especially as a result of external pressures and self-imposed expectations [22].

**Fig 1:**
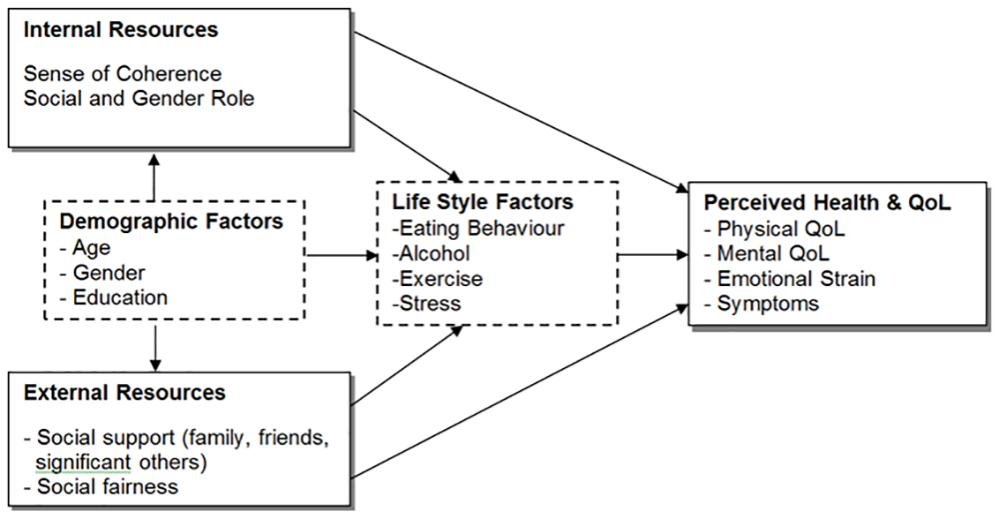
Conceptual model of perceived health and quality of life.

Empirical research has established the salutogenic model as a health promoting resource that improves resilience and develops a positive subjective health state and QoL. The salutogenic approach is an ideal concept for cross-cultural health studies in public health and health promotion, since it is applicable in different cultures at individual, group and societal levels [9]. So far, existing cross-cultural health research lacks a direct comparison between Western and Eastern cultures. The aim of the study was to compare internal and external resources, life style factors, perceived health and QoL in two diverse cultural settings (Japan and Austria) and to determine associations among these factors based on a theoretical model (Fig 1).

We investigated to what extent perceived health and QoL is predicted by internal and external resources, sociodemographic variables, and life style factors. Determinants of health were based on a salutogenic health model including relevant outcomes in terms of health perception and QoL, as well as, predictors that have been shown to be different in various cultures [23].

## Methods

### Ethics Statement

Ethical approvals were obtained from the Karl Franzens University Graz (GZ. 39/36/63) and from Graduate School of Human Development and Environment of Kobe University (No. 70). The study was conducted according to the ethical principles of the Declaration of Helsinki.

### Study Participants

An ad hoc sample of students from one University in Austria and two Universities in Japan were invited to participate. Students from all faculties received an information sheet with a description of the study indicating that participation was voluntary and that their information would be kept confidential by using code numbers. Consenting students filled out the questionnaire package and returned it in an envelope to ensure anonymity. A total of 881 students agreed to participate in the study.

### Data Collection and Measurements

Data were collected simultaneously in Japan and Austria using a battery of self-report questionnaires. For the cross-cultural comparison instruments were chosen which were validated in both countries.

The *Sense of Coherence Scale* 13-item (SOC-13) short form was used to measure sense of coherence. The SOC-13 scale is a valid, reliable and cross culturally applicable instrument which has been widely used in Western and Eastern countries. The scale was validated in Japanese [24] and German populations [25]. Stability of factor structure and predictive validity was assessed in a sample of Japanese undergraduate students [26]. This measure consists of three components: comprehensibility, manageability, and meaningfulness which are equally weighted. The items were rated on a seven-point Likert scale with higher scores indicating a stronger SOC. The total score ranges from 13 (minimum score) to 91 (maximum score). The scale has acceptable psychometric properties with Cronbach’s alpha coefficient in both cultures [24–26]. In this study the Cronbach’s alpha coefficient was 0.81 for the Austrian sample, and 0.61 for the Japanese sample.

*Social and Gender Role Scale* was used to assess attitudes and beliefs towards social and gender role using five statements: 1. General: “*Husband and wife should share their duties at home and at their jobs”; 2*. Mother's role: “*It is the mother's responsibility to care for the children”;* 3. Father's role: “*A father should spend a lot of time with the children during the week*, *not only on weekends”*; 4. Wife's role: “*A married woman should mainly care for her family not for her job”;* 5. Husband’s/partner’s role: “*A husband/partner should be the main financial resource for a family”* [27]. Respondents were asked to indicate how much they agree to these statements on a scale from 1 (“do not agree at all”) to 5 (“completely agree”). Scores range from 5 to 25 with higher scores indicating more traditional attitudes and beliefs towards gender roles. The five statements in English language were translated into Japanese and German respectively by translators (native speakers in the target language) and back-translated. The back-translations were than compared with the original English version to ensure cross-cultural equivalence and to avoid translation errors. Cronbach’s alpha coefficient for the Austrian sample was 0.73 and for the Japanese sample 0.65.

The *Multidimensional Scale of Perceived Social Support (MSPSS)* available in Japanese and German was used to assess the level of perceived social support [28]. The 12-item self-report inventory includes three subscales: support from family, support from friends, and support from significant others. Respondents indicate their agreement with each item on a 7-point response scale from *disagree strongly* (1) to *agree strongly* (7). Higher scores indicate a high level of social support. The psychometric properties were extensively tested in college students. The MSPSS has good internal reliability, and the factor analysis confirmed the three subscale structure [29]. In the present sample Cronbach’s alpha coefficients for the subscales were: family support for Austria 0.93; for Japan 0.90; support from friends for Austria 0.93, for Japan 0.92; support from significant others for Austria 0.97, and for Japan 0.92.

The *Dutch Eating Behaviour Questionnaire (DEBQ)* consists of 33 items comprising three subscales: (a) *Emotional Eating* (13 items), with questions such as “Do you have the desire to eat when you are irritated?”; (b) *External Eating* (10 items), with questions such as “Do you eat more than usual when you see others eating?”; and (c) *Restraint Eating* (10 items) with questions such as “Do you deliberately eat less in order not to become heavier?”. Translations are available in German and Japanese. The psychometric properties were originally tested in samples of obese and non-obese subjects, male and female samples [30]. The three item scale structure was confirmed in the German version of the DEBQ [31]. Respondents indicate their agreement with each item on a 5-point response scale from *disagree strongly* (1) to *agree strongly* (5). Cronbach’s alpha coefficients of the scales range from .80 to .95 indicating high internal consistency and factorial validity. The Cronbach’s alpha coefficients for the present sample were for restrained eating 0.75 for Austria, 0.74 for Japan, for emotional eating 0.74 for Austria, 0.84 for Japan, and for external eating 0.66 for Austria and 0.46 for Japan.

The *SF-12 Health Survey* is a brief, reliable measure of overall health status used to measure two domains: physical health and mental health including 12 items with 3 and 5 point Likert scales [32]. Higher scores indicate a better physical or mental health state. The SF-12 has been cross-culturally validated in several languages including German and Japanese. The SF-12 is an ideal instrument for comparative research in a Western and in an Eastern culture with sufficient evidence for the internal consistency. The reliability of all SF-12 scales was 0.88 [33].

*The Cross-cultural Health Survey* previously used in a comparative cross-cultural health project in Central Europe [34] was adapted for this study. Health indicators were defined by theory-based and internally consistent sets of items. The survey comprised questions concerning socio-demographics, life style factors, and perceived health (emotional strain and symptoms).

- Socio-demographics: gender, age, years of education, living situation (living alone, in students dormitory, with friends in shared apartment, with partner, with family or relatives), perceived social fairness (“Compared to others do you think you get a fair amount of money?” 1 = much less, 2 = less than others, 3 = a fair amount, 4 = more than others, 5 = do not know).
- Life style factors: exercise/sports (hours/minutes per week), alcohol consumption (frequency), level of stress (assessed on linear self-assessment analogue scales 0–100).
- Emotional strain was assessed using 4 items (In the evening *“your daily work does not go out of your mind”*, *“you feel tired or exhausted”*, *“you feel unsatisfied or depressed”*, *“you need to go to bed early”*) scored on a 4 point Likert scale (1 = often to 4 = never) with higher scores indicating less strain.
- Symptoms: headache, heart problems, shortage of breath, sensitive stomach, nervousness, chest pain, pain in the neck or shoulders, back pain, difficulty with concentration, sleeping problems, fatigue/lack of energy, pain in joints or extremities were assessed on a 5-point Likert response scale from 1 = “almost every day” to 5 = “never”. A sum score was established ranging from 12 to 60 with a low score indicating a poorer health state (more symptom experience).

For this study culturally sensitive measures were carefully selected that have been validated in Eastern and Western countries.

### Statistical analysis

To analyse the difference and variation between Austria and Japan covariance analyses were conducted adjusting the samples for age, gender and education. Health indicators were defined by theory-based and internally consistent sets of items. Internal consistency was evaluated by means of Cronbach’s alpha coefficient. Cronbach’s alpha values of 0.70 or higher were considered as acceptable. The data from Austria and Japan were merged and analysed in a universal approach in order to explore for cultural effects. Descriptive statistics were used to analyse the variables by country and by the target samples within each country. Group differences between the countries were calculated using covariance analysis adjusting for age, gender and education. Multiple linear regression models were conducted separately for the Austrian and Japanese sample. QoL (SF-12 physical and mental), perceived health (emotional strain and symptoms) were included as dependent variables. Gender, age, years of education, SOC, social support (significant others, friends, family), gender role, social fairness, eating behavior (restrained, emotional, external), life style factors (alcohol, stress, exercise) as predictor variables. For each model the unstandardized coefficient (Beta), the standard error, the standardized beta coefficient (ß), the explained variance (R-square) and the F-value were calculated. All reported significance levels are p<0.05. The data were analysed using SPSS (version 22).

## Results

Socio-demographic characteristics are shown in Table 1. The sample consisted of 421 students from Austria and 460 students from Japan. They were very interested in the study and the response rate in both samples was very high with no refusals. The mean age in the Austrian sample was 22.13 years compared to 18.89 years in the Japanese sample. Austrian students had more years of education compared to Japanese students. Most participants studied health related sciences (Austria 48.5% vs. Japan 39.3%), followed by technical subjects (Austria 28.5% vs. Japan 21.5%), or economy (Austria 24.0% vs. Japan 22.8%). In the Austrian sample 15.0% of the participants studying other subjects were also included. In Austria 238 (56.3%) were female students and 182 (43.2%) were male students; in Japan 201 (43.7%) were female students and 259 (56.3%) were male students.

**Table 1:** Characteristics of participants.

Variable	AustriaMean		SE	Japan Mean		SE	p
**Sociodemographics**							
Age	22.13			18.89			
Education (years)	15.13			12.36			
% females		56.3			43.7		
% males		43.2			56.3		
**Life style factors**							
Sports (days/ week)	2.45		0.09	3.73		0.13	< .001
Stress level (0–100)	51.00		1.47	52.36		1.39	.550
Restrained Eating	22.63		0.34	32.03		0.31	< .001
Emotional Eating	29.82		0.44	35.26		0.42	< .001
External Eating	21.94		0.28	27.25		0.26	< .001
**Internal Resources**							
Gender/Social Role	10.96		0.18	11.89		0.17	.001
Sense of Coherence	61.74		0.55	52.55		0.52	< .001
**External Resources**							
Social support—Family	24.40		0.32	20.36		0.30	< .001
Social support—Friends	24.83		0.30	19.57		0.29	< .001
Social support—Sign. Others	25.15		0.31	19.91		0.29	< .001
**Perceived Health and QoL**							
Emotional strain	8.78		0.12	9.23		0.11	.006
Symptoms	25.56		0.47	24.80		0.44	.298
QoL Physical (SF-12)	54.02		0.38	50.27		0.36	< .001
QoL Mental (SF-12)	54.21		0.38	50.29		0.36	< .001

QoL = quality of life; SE = standard error, Adjusted for age, gender and education

Concerning life style factors Japanese students do significantly more exercise compared to their Austrian counterparts. The level of stress is similar in both samples. Japanese students showed significantly higher mean scores on the DEBQ indicating a higher degree of restrained, emotional and external eating compared to Austrian students. Table 1 shows the comparison of internal resources, external resources, perceived health and QoL. Japanese students showed significantly higher mean scores on the Social and Gender Role Scale indicating more traditional attitudes and beliefs compared to Austrian students. The mean score for SOC was significantly higher in the Austrian sample compared to the Japanese sample. Concerning external resources Austrian students report having more social support from family, friends and significant others compared to Japanese students. Emotional strain and symptoms were significantly higher in Japan than in Austria. The mean scores for the physical and mental QoL domains (SF12) were significantly lower in the Japanese sample compared to the Austrian sample.

Separate multiple linear regression analyses were conducted to identify predictors for physical and mental QoL, as measured by the SF12, as well as, for emotional strain and perceived symptoms. Tables 2 and 3 show the results of the regression analyses for Austria and Japan, respectively. For the physical domain of QoL as measured by the SF-12 only exercise in the Austrian sample and external eating in the Japanese sample were significantly related to the physical QoL domain. All other variable were not related. In both models less than 10% of the variance was explained. For the mental domain of QoL (SF-12), emotional strain, and symptoms, the linear regression models accounted for 48%, 42%, 40% of the variance in Austria and 40%, 35% and 22% in Japan, respectively. SOC was the strongest predictor in both groups associated with mental QoL, mental strain and symptoms (standardized ß coefficients in Austrian 0.47, 0.27, -0.38 and Japanese 0.37, 0.29, -0.34). SOC showed no relationship to physical QoL in either group.

**Table 2:** Associations between perceived health, quality of life and predictor Variables—Austrian sample.

	Physical QoL		Mental QoL		Emotional Strain		Symptoms	
Variables	ß*	p	ß	p	ß	p	ß	p
Gender	.030	.651	-.047	.348	-.243	-.001	.104	.053
Age	-.086	.123	-121	.004	-.136	.002	.104	.020
Education	.010	.848	-024	.557	-.049	.250	-.050	.253
Sense of Coherence	.023	.691	.468	< .001	.268	< .001	-.379	< .001
Significant others	-.011	.933	-.186	.063	.049	.642	.231	.025
Friends	.142	.302	-.070	.494	.053	.631	-.054	.619
Family	-.072	.712	.354	.016	.019	.906	-.212	.167
Gender role	.003	.964	.017	.648	.096	.029	-.049	.268
Social fairness	.091	.089	-.040	.317	-.013	.764	-.042	.321
Restrained eating	.112	.297	-.121	.129	-.194	.020	.074	.388
Emotional eating	.035	.730	.023	.759	-.006	.939	.185	.027
External eating	-.039	.696	-.089	.234	.082	.299	-.056	.488
Alcohol	-.073	.183	-.053	.190	-.158	< .001	.045	.297
Stress	.002	.977	-.211	< .001	-.253	< .001	.145	.001
Exercise	.137	.014	.079	.055	.077	.077	-.124	.005
R^2^	7%		48%		42%		40%	

QoL = quality of life;

*ß indicates standardized ß coefficient.

**Table 3:** Associations between quality of life, emotional strain, symptoms and predictor variables—Japanese sample.

	Physical QoL		Mental QoL		Emotional Strain		Symptoms	
Variables	ß*	p	ß	p	ß	p	ß	p
Gender	.065	.482	-.011	.883	-.040	.614	.008	.928
Age	-.031	.782	.027	.759	-.204	.028	-.161	.117
Education	.014	.898	-100	.252	.111	.226	-.039	.699
Sense of Coherence	.066	.441	.365	< .001	.291	< .001	-.341	< .001
Significant others	-.116	.605	.142	.428	-.043	.821	.143	.486
Friends	.138	.470	.100	.515	.116	.483	-.025	.890
Family	.093	.754	-.157	.510	.040	.878	-.096	.732
Gender role	.001	.990	-.014	.832	-.019	.780	-.055	.469
Social fairness	< .001	996	-.101	.104	.077	.230	-.021	.766
Restrained eating	-.157	.217	.018	.863	.014	.898	.152	.190
Emotional eating	-.072	.599	-.031	.778	-.001	.997	-.059	.642
External eating	.296	.014	-.022	.814	.106	.284	-.057	.603
Alcohol	.003	.968	-.041	.537	.008	.911	-.128	.100
Stress	-.057	.506	-.377	< .001	-.329	< .001	.157	.045
Exercise	-.068	.387	.052	.406	-.076	.248	-.080	.269
R^2^	8%		40%		35%		22%	

QoL = quality of life;

*ß indicates standardized ß coefficient.

In the Austrian sample, age and stress were negatively related. SOC and family support were positively related to mental QoL. In the Japanese sample stress was negatively related and SOC positively related to mental QoL. Significant predictors for emotional strain were female gender, older age, lower SOC, less traditional gender and social role patterns, more restrained eating, more alcohol intake, and more stress explaining 42% of the variance in Austrian students. In the Japanese sample only older age, lower SOC and more stress were identified as significant predictors explaining 35% of the variance. In Austria and Japan perceived symptoms were negatively associated with SOC and positively associated with stress. Only in the Austrian sample, older age, more support from significant others and higher emotional eating were significantly related to symptoms.

## Discussion

In this cross-cultural project we investigated the relationship between internal and external resources, life style factors, perceived health and QoL in an Eastern and a Western culture. This study was based on the Antonovsky’s salutogenic framework which provides a good theoretical foundation for health promotion. We conducted the study in homogenous cohorts of student, in two diverse cultures, Japan and Austria. Study participants were widely comparable in terms of their socioeconomic status which allows detecting cultural differences by minimizing confounding variables such as income, education, or employment. However, we found that Japanese students were on average three years younger with three years less education. This is due to the fact that in Japan students have their regular University entrance examination after high school at the age of 18 years. The Austrian education system is more flexible. Students attending the University of Applied Sciences can enter after high school, but a considerable number of students have a college degree or have had a job before entering University.

The results showed significant cross-cultural differences in most of the areas studied. Japanese students exercise more extensively when compared to Austrian students, although in both countries the Universities offer a variety of opportunities for sports. Students attending the University of Applied Science in Austria have a tight time schedule that does not allow much time for leisure activities. This is a common complaint from students in Austria. Concerning eating behavior significant differences were found between the Japanese and the Austrian samples. On all three DEBQ scales (emotional, external, and restraint eating) Japanese students scored higher. In line with other studies we found that eating behavior is strongly influenced by cultural effects [35]. Patterns such as eating in response to emotional arousal states, eating in response to external food cues, and restrained eating to control their weight seems to be more prevalent with Japanese students, in this study. Katou et al. [36] reported a similar pattern and a correlation between eating behaviour and body mass index in the Austrian sample but not in the Japanese sample. Even if they are very slim, Japanese students are not satisfied with thier body image and strongly control their eating behaviour. Restrained eating is also associated in Japanes students with less snacking behaviour [37].

Concerning the social and gender role, Japanese students showed more traditional attitudes and beliefs compared to Austrian students. This is not surprising since Eastern cultures tend to value traditional gender role expectations and social role conceptions, which are less pronounced in Western cultures. Although the study sample comprised young and well educated people, the attitudes are deeply rooted in the traditional society of Japan. Women in Japan still have the main responsibility for the household, allowing men to devote themselves to their work [38]. Modern Japanese gender roles revolve around their vertical society where someone’s identity is a part of their group identity. Social support has been recognised to greatly influence collective and personal well-being [39]. It is one of the most effective means by which individuals can cope with stressful events. People from different cultural backgrounds may be affected by support from others differently, even if they have equally supportive social networks. In this study Austrian students reported having more social support compared to Japanese students. This may be explained by the fact that in the more individualistic cultures, such as in Austria, people may ask for social support with relatively little caution. They share the cultural assumption that individuals should proactively pursue their well-being and that others have the freedom to choose to help. In contrast, in the more collectivistic cultures like Japan, people may be more hesitant when asking others for help. Japanese share the cultural assumption that individuals should not burden their social networks [40]. Similar to other studies it appears that the tendency not to seek social support is a quite general phenomenon in Asian countries. Asians may seem to evaluate social support and support seeking differently due to the belief that one should not have to ask for support because people should anticipate their need for support and provide for it before support is explicitly sought.

In this study Japanese students showed lower levels of social support, lower scores on the SOC scale, and on the physical and emotional domain of QoL, and more emotional strain compared to Austrians. The different responses may be explained by the fact that health perceptions and subjective well-being are partly shaped by cultures. The experience of health and well-being is dependent upon the individual appraisal of external and internal resources and values in the context of culture, which may result in a response bias in cross-cultural self-report studies [16, 41]. Previous research has shown that in European-American cultures, well-being tends to be defined as personal achievement, whereas in East Asian cultures it tends to be defined as realization of social harmony [42]. Kim et al. [40] found that Asians are more reluctant to explicitly ask for support from others than Europeans or Americans. Asians are more concerned about the potentially negative relationship consequences of seeking support, such as disrupting group harmony. People from different cultural backgrounds may utilize and be affected by support from others differently, even if they have equally supportive social networks. The lower scores reported by Japanese students can be explained by the fact that conventionally used measures may be in favor of independent goals such as self-enhancing tendencies which are more related to Western cultures [43]. Although the mean scores in the Japanese sample were generally lower we identified similar determinants of health and QoL between two diverse cultures. Independent of the cultural background of the study participants SOC seems to be the strongest predictor for mental QoL but not for physical QoL. As in our study, Biro et al. [19] found that SOC was a strong explanatory variable for psychological distress related to perceived health in medical students. Von Bothemer et al. [18] found a positive correlation between perceived health and SOC in female students but not in male students.

Several limitations of this study should be noted and some caution is warranted when interpreting the results of this study. Although we have carefully selected culturally sensitive measures the internal consistency for some subscales was poor. In the Japanese sample more scales were below the acceptable alpha values (three below 0.70 and one below 0.50) than in the Austrian sample (only one below 0.70). However, most of the subscales had acceptable Cronbach alpha values of 0.70 or higher. Values between 0.70–0.80 are regarded as satisfactory for comparing groups [44]. Although only cross-culturally validated measures were used an inherent cultural bias cannot be ruled out. We used a psychometrically sound scale to measure social support with strong factorial validity and good internal reliability. The MSPSS may not have been sensitive enough to differentiate between students with good versus poor social support and we could not confirm the predictive power of social support on mental QoL. However, this is in line with other researchers reporting that perception of emotional support sometimes has no positive effects on subjective well-being [45]. Further research is required to study how the effect of perceived social support on mental well-being is moderated by culture. Previous studies showed that Japanese people report relatively poor subjective well-being, although interpersonal trust is significantly related to better QoL [46]. It seems that Austrian students may have the tendency to report better scores on the questionnaires than Japanese students. Another limitation of the study is that health was assessed only by self-reports and not by physical examinations. Subjective well-being and perceived health based on subjective reports are important predictors of mortality [47].

Despite these limitations we conclude that higher SOC and less stress are strongly associated with better QoL and perceived health in Austria as well as in Japan. SOC seems to be crucial predictor for stress and emotional health independent of the cultural context. A major challenge of cross-cultural research is to understand that there are considerable cultural differences in how people view the self and their relationships with others. In this study we compared health related factors in students living in an individualistic society in Austria with a sample of young students living in a collectivistic society in Japan. Given the diverse cultural settings the findings of the results of this study contribute to improve our understanding of perceived health and QoL, and the extent to which it is individually, socially, or culturally determined. The results support the development of cross-culturally applicable health indicators which are important for health promotion, health policies and health monitoring in the respective country and culture [48].
